# Comparison between magnetic resonance and ultrasound-derived indicators of hepatic steatosis in a pooled NAFLD cohort

**DOI:** 10.1371/journal.pone.0249491

**Published:** 2021-04-01

**Authors:** Cayden Beyer, Chloe Hutton, Anneli Andersson, Kento Imajo, Atsushi Nakajima, Dustin Kiker, Rajarshi Banerjee, Andrea Dennis

**Affiliations:** 1 Perspectum, Oxford, United Kingdom; 2 Department of Gastroenterology and Hepatology, Yokohama City University Graduate School of Medicine, Yokohama, Japan; 3 Texas Digestive Disease Consultants, Dallas, Texas, United States of America; Kaohsiung Medical University, TAIWAN

## Abstract

**Background & aims:**

MRI-based proton density fat fraction (PDFF) and the ultrasound-derived controlled attenuation parameter (CAP) are non-invasive techniques for quantifying liver fat, which can be used to assess steatosis in patients with non-alcoholic fatty liver disease (NAFLD). This study compared both of these techniques to histopathological graded steatosis for the assessment of fat levels in a large pooled NAFLD cohort.

**Methods:**

This retrospective study pooled N = 581 participants from two suspected NAFLD cohorts (mean age (SD) 56 (12.7), 60% females). Steatosis was graded according to NASH-CRN criteria. Liver fat was measured non-invasively using PDFF (with Liver *MultiScan*’s Iterative Decomposition of water and fat with Echo Asymmetry and Least-squares estimation method, LMS-IDEAL, Perspectum, Oxford) and CAP (FibroScan, Echosens, France), and their diagnostic performances were compared.

**Results:**

LMS-IDEAL and CAP detected steatosis grade ≥ 1 with AUROCs of 1.00 (95% CI, 0.99–1.0) and 0.95 (95% CI, 0.91–0.99), respectively. LMS-IDEAL was superior to CAP for detecting steatosis grade ≥ 2 with AUROCs of 0.77 (95% CI, 0.73–0.82] and 0.60 (95% CI, 0.55–0.65), respectively. Similarly, LMS-IDEAL outperformed CAP for detecting steatosis grade ≥ 3 with AUROCs of 0.81 (95% CI, 0.76–0.87) and 0.63 (95% CI, 0.56–0.70), respectively.

**Conclusion:**

LMS-IDEAL was able to diagnose individuals accurately across the spectrum of histological steatosis grades. CAP performed well in identifying individuals with lower levels of fat (steatosis grade ≥1); however, its diagnostic performance was inferior to LMS-IDEAL for higher levels of fat (steatosis grades ≥2 and ≥3).

**Trial registration:**

ClinicalTrials.gov (NCT03551522); https://clinicaltrials.gov/ct2/show/NCT03551522. UMIN Clinical Trials Registry (UMIN000026145); https://upload.umin.ac.jp/cgi-open-bin/ctr_e/ctr_view.cgi?recptno=R000026145.

## Introduction

Non-alcoholic fatty liver disease (NAFLD) is characterized by excessive liver fat in the absence of heavy alcohol consumption, and is commonly associated with metabolic diseases such as Type 2 diabetes and obesity [1,2]. For some people NAFLD progresses on to NASH (Non-alcoholic steatohepatitis), a more serious condition, which can cause liver fibrosis, and eventually cirrhosis, the most severe stage of NAFLD.^1^ While the precise prevalence of NAFLD is unknown due to relatively unreliable diagnostics and absence of widespread testing, it has been estimated to affect 25% of the worldwide population.^2^ Modelling has forecasted that global NAFLD cases will rise to nearly 100.9 million by 2030; a 33.5% increase from 2015 [3]. With NAFLD arising as the most common liver disease worldwide, so are the demands for reliable diagnoses [4].

Liver biopsy is the current gold-standard for NAFLD diagnosis, but this invasive procedure has high degrees of variability and sampling error in addition to carrying associated risks and costs [5,6]. Liver biopsies have proved to be subjective with up to 50% of pathologist reads resulting in disagreement [7]. With these well-known limitations of liver biopsy and the increasing need to perform liver health assessments, a number of non-invasive imaging techniques have emerged as important biomarkers for quantifying liver fat in both patient care and clinical trials [8].

There is a growing consensus that MRI proton density fat fraction (PDFF) is currently the most accurate, precise, and reproducible way to quantify liver fat non-invasively [9–12]. This technique uses MRI sequences that are available on most scanners with advanced image processing to measure the ratio of fat protons to total fat and water protons, while correcting for confounds resulting from the MRI imaging process. In particular, an MRI PDFF technique known as IDEAL (Iterative Decomposition of water and fat with Echo Asymmetry and Least-squares estimation) has addressed the impact on the MRI signal of iron in the liver and the multi-spectral nature of liver fat [13–15]. MRI PDFF methods have been shown to correlate well with histological grading of steatosis levels and provide excellent diagnostic accuracy, sensitivity, and specificity [16–22]. However, it is worth noting that many of the studies comparing MRI PDFF with histology have been performed with cohort sizes of less than 200, and with data collected at a single site using a single scanner model.

Recently the IDEAL technique for MRI-based liver fat quantification has been further refined to be robust to variations in how different MRI scanners process data [23]. These developments have resulted in a technique, referred to here as LMS-IDEAL, that quantifies liver fat in a standardised way across different manufacturers and field strengths. LMS-IDEAL is integrated in the multiparametric MRI technology, Liver *MultiScan*^®^ (Perspectum, UK), a diagnostic aid to quantitatively characterise liver tissue in terms of fat, iron, fibrosis and inflammation [24–26]. Liver *MultiScan*’s standardised measurements allow data from larger cohorts, comprising participants from different studies, locations and scanners to be combined in a single analysis.

Another method available for measuring liver fat is the ultrasound-derived Controlled Attenuation Parameter (CAP^™^). CAP is a parameter that measures the attenuation, or reduction in amplitude, of ultrasound waves travelling through a liver [27]. The measurement is performed using the ultrasound-based technology FibroScan^®^ [Echosens, Paris, France] [28]. Although CAP has been shown to work well for the detection of significant hepatic steatosis, results from different studies suggest discordance between CAP and histology scores, and in particular, the diagnostic accuracy is impaired by an increased BMI as well as presence of NASH, NAFLD, and Type 2 diabetes [29–36].

In a retrospective analysis of a large group of 580 participants with high liver fat, pooled together from two different clinical studies and four different scanner models, we set out to assess the relative diagnostic accuracies of LMS-IDEAL and CAP to discriminate between different levels of histologically graded steatosis in patients with suspected NAFLD/NASH.

## Methods

### Design and study participants

This study was a retrospective analysis of N = 581 participants (mean age of 56 yrs (SD: 12.6); 60% female) pooled together from two independent studies, each referred to here as a ‘parent study’. The first parent study (study 1, N = 434) was an interventional NASH clinical trial in a US population recruited from secondary care between May 2018 and January 2019, for which inclusion criteria included being aged between 18 and 75, and having an MRI liver fat measure, LMS-IDEAL ≥ 10%. These participants went on to have biopsies, and although they were screened for evidence of NASH for the treatment trial (where evidence included a NAS (NAFLD Activity Score) ≥ 4 with a score ≥ 1 in each component (steatosis, lobular inflammation, and ballooning), and biopsy-scored fibrosis stage 1, 2, or 3), all pre-screening data (i.e. participants without biopsy) were also used in the correlational analysis to ensure a good spread of true liver fat levels in this analysis. The second parent study (study 2, N = 146) is an ongoing prospective cross-sectional study to assess NASH prevalence in the Japanese population recruited through secondary care and included participants with evidence of high liver fat based on imaging results acquired between January 2019 and February 2020; all of whom had liver biopsy on suspicion of NASH [37]. The participant inclusion criteria included being aged between 18 and 80 and having an MRI liver fat measure ≥ 5.2% (note this screening measure used a different post-processing technique to LMS-IDEAL (IDEAL IQ, GE Healthcare), but the two measures were in excellent agreement, r = 0.94; all data used in subsequent analyses were reprocessed with LMS-IDEAL) or ultrasound-based liver fat measure, CAP ≥ 236 dB/m [38]. These participants went on to have biopsies. The exclusion criteria included history of alcoholism, evidence of other chronic liver disease and contraindication for MRI. Both parent studies were conducted in accordance with the ethical principles of the Declaration of Helsinki 2013 and all participants gave written informed consent. Study 1 was approved by the institutional review departments at each of the included 15 clinical trial sites in the United States of America and registered with clinicaltrials.gov (NCT03551522). Study 2 was approved by the Ethics Committee of Yokohama City Hospital and registered with UMIN clinical trials registry (UMIN000026145).

From the pooled data, 451 had baseline liver biopsy results, graded using steatosis Clinical Research Network (CRN) score [39], by a pathologist blinded to patient characteristics and non-invasive assessment data. Biopsy scores used for the analysis were those collected as part of the two independent studies and were not re-read centrally. High body mass index (BMI) has been reported to be a limitation for successful CAP measurements [36], as such BMI has been reported for all cases where the data were available (N = 208, study 1; N = 146, study 2). Full demographic details are presented in Table 1.

**Table 1:** Baseline patient characteristics.

	All (n = 580)	Study 1 (434)	Study 2 (n = 146)
**Age (yrs; mean [SD])**	56 [12.6]	55 [12.2]	60 [13.1]
**Sex (F, %)**	350 (60%)	293 (68%)	57 (39%)
**BMI (weight/height^2^; median [IQR])**	31.39 [26.8–36.8]	34.16 [29.0–39.2, n = 208]	28.28 [25.5–32.3]
	**N = 451**	**N = 305**	**N = 146**
**Fibrosis (n,%)**			
F0	44 (9.8)	39 (12.8)	5 (3.4)
F1	110 (24.4)	79 (25.9)	31 (21.2)
F2	140 (31.0)	108 (35.4)	32 (21.9)
F3	118 (26.2)	66 (21.8)	52 (35.6)
F4	39 (8.6)	13 (4.3)	26 (18.8)
**Ballooning (n,%)**			
B0	134 (29.7)	89 (29.2)	45 (30.8)
B1	176 (39.0)	98 (32.1)	78 (53.4)
B2	141 (31.3)	118 (38.7)	23 (15.8)
**Lobular Inflammation (n,%)**			
I0	3 (0.7)	2 (0.7)	1 (0.7)
I1	200 (44.3)	129 (42.6)	71 (48.6)
I2	225 (49.9)	161 (52.5)	64 (43.8)
I3	23 (5.1)	13 (4.3)	10 (6.8)
**Steatosis (n,%)**			
S0	8 (1.8)	1 (0.3)	7 (4.8)
S1	224 (49.7)	156 (51.5)	68 (46.6)
S2	139 (30.8)	89 (29.2)	50 (34.2)
S3	80 (17.7)	59 (19.0)	21 (14.4)
**LMS-IDEAL PDFF (mean %; [SD])**	16.5 [9.0]	18.2 [9.2]	11.6 [6.3]
**CAP (mean [SD])**	322.5 [51.5)]	332.2 [48.6]	294.6 [47.7]

### Magnetic resonance imaging

Each participant had a liver MRI examination at one of the 16 different clinical trial sites located in the United States of America or Japan, on either a 1.5T and 3T Siemen’s scanner (Siemens Healthineers, Erlangen, Germany) or a 1.5T GE scanner (GE Healthcare, Waukesha, WI). Each site had previously received full training and passed image quality assurance processes for the MRI data to be included in the trial.

**Fig 1:**
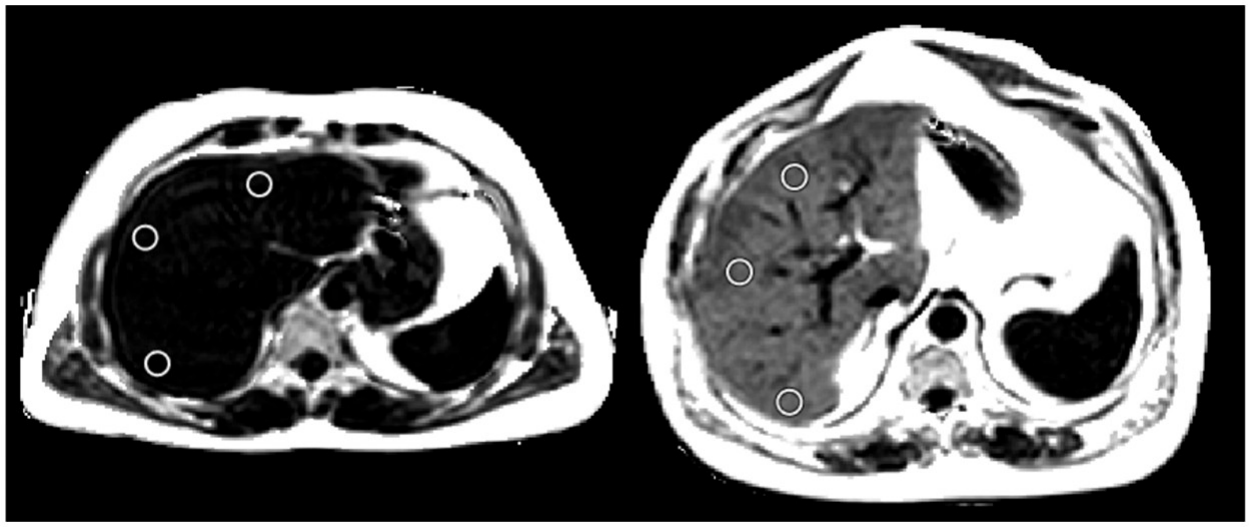
Example LMS-IDEAL PDFF maps.

MRI scanning was performed using Liver *MultiScan* image acquisition protocols [24–26]. For LMS-IDEAL PDFF measurements, four sets of transverse images of the liver were acquired at the level of the portal vein. Anonymised MRI data were processed and analysed centrally by expertly trained image analysts using Liver *MultiScan*. The processing included the calculation of LMS-IDEAL PDFF maps of the liver (measured in %) using proprietary algorithms based on the multispectral IDEAL approach, which is robust to MRI-related confounds [23]. Analysis included the calculation of LMS-IDEAL measures from the median value over three manually placed regions of interest in the right lobe of the liver, avoiding image artefacts and vessels (Fig 1).

### Controlled attenuation parameter

Each participant had a CAP measurement (measured in decibels per meter, dB/m) using an ultrasound-based FibroScan examination. The FibroScan device was used to acquire VCTE^™^ (Vibration Controlled Transient Elastography) liver stiffness measures (LSM) with either a 3.5 MHz M-probe and/or 2.5MHz XL-probe, dependent upon suitability (waste-hip circumference and BMI), using the automatic probe selection tool embedded within the FibroScan operating software [28]. CAP measurements were calculated from the same ultrasound signals [27] only when LSM (not reported here) were reliable, where reliability was defined by LSM having at least 10 valid shots and a success rate of ≥60%.

### Statistical analyses

Statistical analyses were performed using R Studio version 3.6.0 (R Core Team, 2016. R: A Language and Environment for Statistical Computing, Vienna, Austria. https://www.R-project.org/). Spearman’s rank correlation coefficient (r_s_) was calculated to evaluate the correlations between LMS-IDEAL and biopsy steatosis grades, CAP and biopsy steatosis grades, and CAP and LMS-IDEAL. Pearson’s correlations (r) were used to explore the relationships between BMI and both CAP and LMS-IDEAL. Correlation results were considered to be significant with a p-value <0.01.

Receiver operating characteristic (ROC) curves [40] were calculated to evaluate the diagnostic accuracy of LMS-IDEAL and CAP to classify the data according to biopsy steatosis grades ≥1, ≥2, and ≥3. Subsequently, the area under the receiver operating characteristic (AUROC) curves and 95% confidence intervals (CI) were calculated and Youden’s index used to calculate the optimal thresholds respectively for LMS-IDEAL and CAP to differentiate between the different steatosis levels. Sensitivity, specificity, positive, and negative predictive values (NPV, PPV) were calculated at the thresholds associated with the Youden’s index to estimate the performance of the two non-invasive liver fat measures. Whilst this was a retrospective analysis, in order to ensure the study was sufficiently powered to assess the diagnostic accuracy of the two methods, a power calculation was performed to identify the optimal sample size for discriminating those with steatosis ≥2. Assuming a low area under the curve of 0.6, with 90% power, and an equal number of cases (S≥2) and controls (S<2), a minimum of 330 participants were required.

## Results

### Missing data/technical failure rates

Out of the total of 580 participants included in the study, LMS-IDEAL failed in one case (0.2%) and CAP was missing in 46 cases (8.0%). Average BMI in the missing versus successful CAP measurements were the same (32.1 versus 32.4 respectively, t = 0.3, P = 0.76), LMS-IDEAL however was significantly lower in the group with missing CAP measurements (13.7 versus 16.7, t = 2.2, P < .05). For the CAP data we do not have the information to confirm whether missing measurements were technical failures or were missing due to CAP not being performed.

### Mean values for LMS-IDEAL and CAP and correlation with biopsy-scored steatosis grades

**Fig 2:**
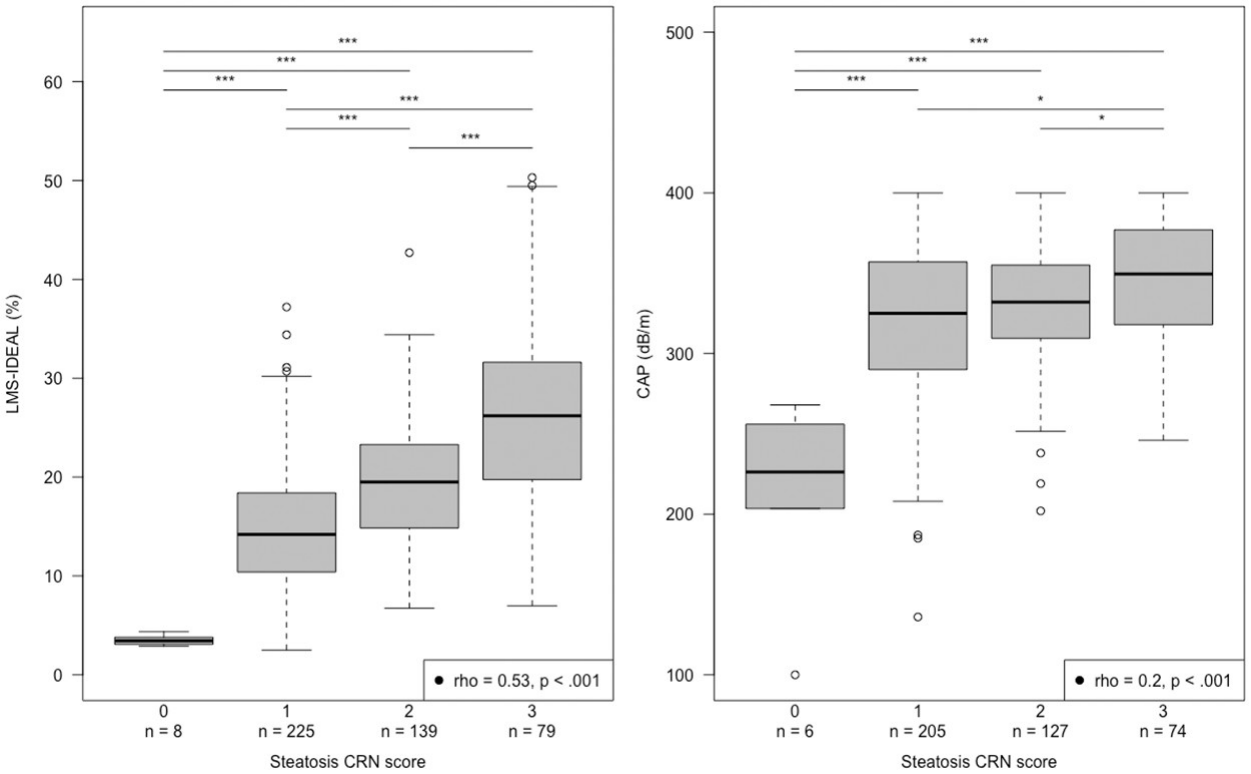
Box plots showing the minimum, median, maximum and interquartile ranges (IQR) values for LMS-IDEAL, and CAP with corresponding steatosis scores. (left) LMS-IDEAL values plotted against steatosis CRN scores, (right) CAP values plotted against steatosis CRN scores.

Mean LMS-IDEAL and CAP measurements for the cohort were 16.4 (%) and 323 (dB/m) respectively. In the subsets of participants with liver biopsy data, the correlation between histologically graded steatosis and LMS-IDEAL was good (r_s_ = 0.53, p < .001) (Fig 2—left), whereas the relationship with CAP was weak (r_s_ = 0.22, p < .001) (Fig 2—right). Correlations between LMS-IDEAL and histologically graded steatosis for the two studies independently were r_s_ = 0.59, p < .001 for study 1 and r_s_ = 0.73, p < .001 for study 2, and between CAP and steatosis were r_s_ = 0.16, p < .01 for study 1 and r_s_ = 0.44, p < .001 for study 2.

Pairwise comparison between each steatosis stage grade revealed significant differences (p < .001) between grades 0 and 1 for both biomarkers, and a continued pattern of significant differences between each grade for LMS-IDEAL (Fig 2—left); CAP however only weakly separated grades 1 from 2 (p < .05) and grades 2 from 3 (Fig 2—right).

**Fig 3:**
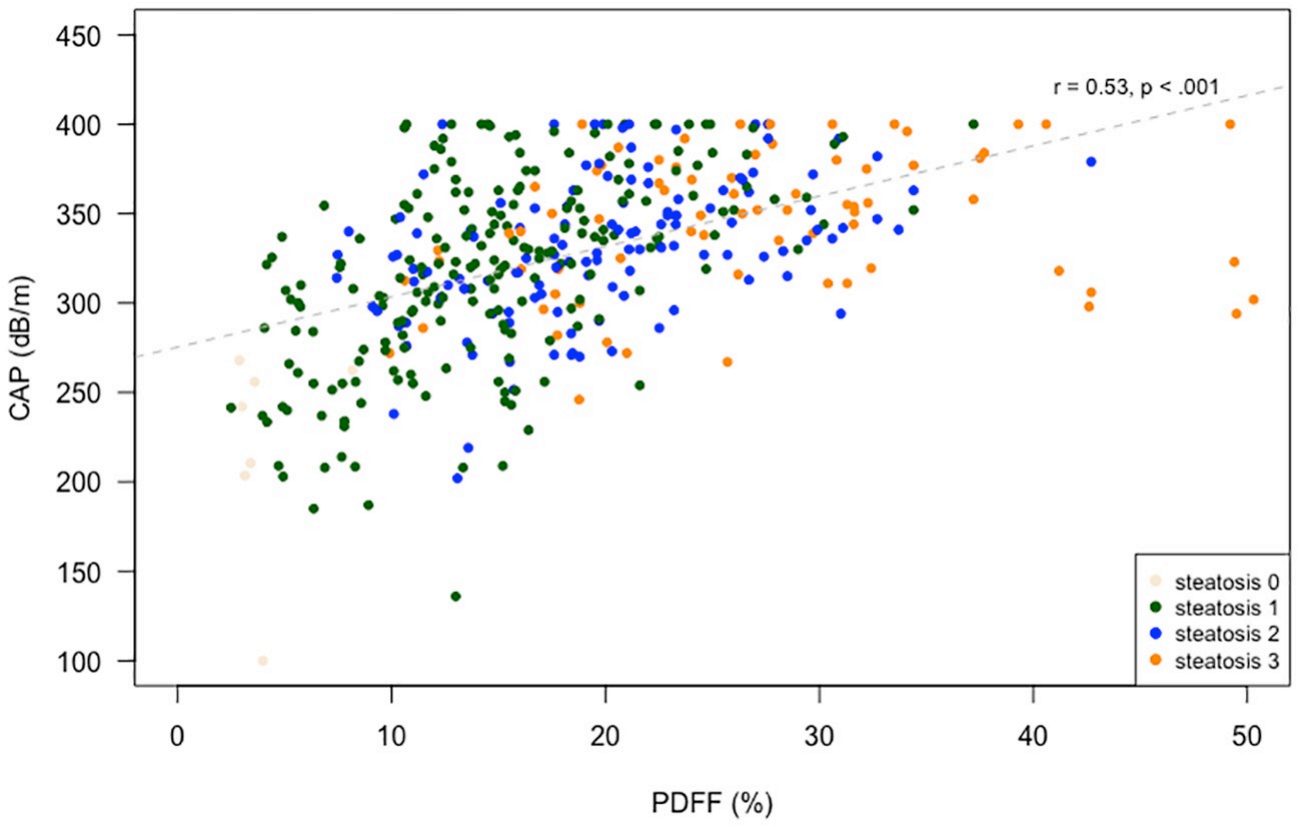
Scatter plot showing CAP values plotted against LMS-IDEAL PDFF values.

Despite the CAP measurement being limited to 400 dB/m, a good correlation between LMS-IDEAL and CAP was also observed (r = 0.53, p < .001), but with a wide spread of values at all fat levels (Fig 3).

BMI data was available for 354 of the 580 participants; of these, 68% (240) had BMI≥28 kg.m^-2^. LMS-IDEAL was weakly correlated with BMI (r = 0.15, P < .01), and CAP more strongly correlated with BMI (r = 0.41, P < .001). Steatosis however was not significantly correlated with BMI (r_s_ = 0.1, P = .09). When the group was sub-divided into those with BMI ≥28 kg.m^-2^, the correlation between BMI and LMS-IDEAL was no longer significant (r = 0.08, P = 0.24), but the correlation between BMI and CAP remained (r = 0.23, P < .001).

### Diagnostic accuracy of LMS-IDEAL and CAP

**Fig 4:**
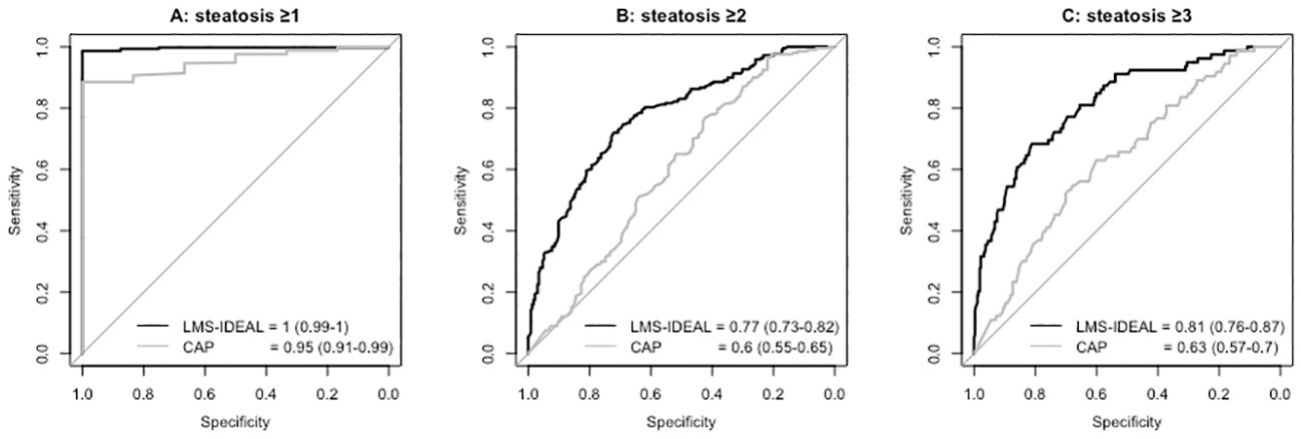
ROC curves illustrating diagnostic accuracies for LMS-IDEAL (black) and CAP (grey). Values represent AUROC (95% CI). (A) LMS-IDEAL and CAP for diagnosing steatosis grades of ≥ 1. (B) LMS-IDEAL and CAP for diagnosing steatosis grades of ≥ 2. (C) LMS-IDEAL and CAP for diagnosing steatosis grades of ≥ 3.

To identify those with steatosis grade ≥1, both biomarkers had good diagnostic accuracy and performance. For LMS-IDEAL, the AUROC = 1.0 (95% CI, 0.99–1) and for CAP, the AUROC = 0.95 (95% CI, 0.91–0.99) (Fig 4A). To distinguish those with more advanced liver fat (steatosis grade ≥ 2), LMS-IDEAL had good diagnostic accuracy, with an AUROC = 0.77 (95% CI, 0.73–0.82). CAP performed significantly (P < .001) worse, with an AUROC = 0.60 (95% CI, 0.55–0.65) (Fig 4B). For distinguishing those with the highest fat (steatosis grade ≥ 3), LMS-IDEAL had good diagnostic accuracy with an AUROC = 0.81 (95% CI, 0.76–0.87), while CAP had significantly (P < .001) lower diagnostic accuracy with an AUROC = 0.63 (95% CI, 0.57–0.70). Diagnostic performance characteristics for discriminating different levels of steatosis, based on thresholds derived from the Youden’s index, are shown in Table 2.

**Table 2:** Diagnostic performance characteristics of LMS-IDEAL and CAP for identifying different steatosis grades.

Steatosis	Fat metric	AUROC	95% CI	Youden’s index threshold	Sens (%)	Spec (%)	PPV (%)	NPV (%)
**≥ 1 (n = 225)**	LMS-IDEAL (%)	1.0	0.99–1.00	4.4	0.99	1.0	1.0	0.57
	CAP (dB/m)	0.95	0.91–0.99	268.5	0.89	1.0	1.0	0.12
**≥ 2 (n = 139)**	LMS-IDEAL (%)	0.77	0.73–0.82	17.5	0.72	0.72	0.70	0.73
	CAP (dB/m)	0.60	0.55–0.65	308.5	0.78	0.41	0.56	0.65
**≥ 3 (n = 80)**	LMS-IDEAL (%)	0.81	0.76–0.87	22.4	0.68	0.81	0.43	0.92
	CAP (dB/m)	0.63	0.57–0.70	337.8	0.61	0.59	0.24	0.87

## Discussion

This retrospective study of a large group of NAFLD participants pooled together from two independent studies demonstrates that liver fat measurements obtained with LMS-IDEAL were more strongly correlated with histology graded steatosis than those obtained using CAP. This study extends the existing literature by showing that LMS-IDEAL was able to accurately diagnose those individuals with lower, as well as more advanced, grades of steatosis according to histology. CAP could accurately diagnose steatosis when the cohort included individuals with lower levels of fat (steatosis ≥1), but diagnostic performance was inferior to LMS-IDEAL for higher levels of fat (steatosis ≥2 and ≥3). This is the first analysis to compare LMS-IDEAL to CAP. LMS-IDEAL uses an advanced post-processing technique called MAGO (magnitude only reconstruction), which resolves the water-fat ambiguity over the entire fat fraction dynamic range without compromising accuracy. Therefore, robust PDFF estimations are enabled where phase data is inaccessible or unreliable, and where hybrid and complex-based methods (typically used by other MRI PDFF techniques) may fail [23]. The direct comparison between LMS-IDEAL and CAP measurements showed a strong correlation, but with a wide spread of values across all fat levels. The results of this study suggest that in NAFLD patients, MRI PDFF measured using LMS-IDEAL can classify different steatosis levels more accurately than CAP, especially at higher steatosis levels. This conclusion is in agreement with other studies reporting that the diagnostic performance of CAP is inferior to MRI PDFF [41,42], with CAP studies even using MRI PDFF as the gold standard for measuring liver fat [12,43–45]. However, to our knowledge this is the largest study to date (more than 500 participants compared with less than 150) directly comparing MRI PDFF and CAP against histology in NAFLD patients, and is the first to pool data from different scanner manufacturers and field strengths.

The optimal LMS-IDEAL and CAP thresholds calculated from Youden’s index based on our data for identifying steatosis levels in the current study were in agreement with those reported in the literature. For example, the minimum and maximum of all the optimal MRI PDFF thresholds reported in the cited studies to identify steatosis grades ≥ 1, ≥ 2 and ≥3 were [min max] = [3.7% 12.5%], [11.3% 17.4%], and [16.7% 26.5%] respectively [16–19,21,41,42]. The minimum and maximum of all the CAP thresholds reported in the cited studies to identify steatosis grades ≥ 1, ≥ 2 and ≥3 were [min max] = 236 dB/m 331 dB/m], [256 dB/m 361dB/m], and [283 dB/m 344 dB/m] respectively [29–35,41,42]. Further studies are required to validate the applicability of specific optimal thresholds for identifying different steatosis grades.

The increasing numbers of people affected by NAFLD means that cost-effective and non-invasive methods for early detection are critical for tackling this disease. In this study, as well as others, CAP was able to detect significant hepatic steatosis, and although it has advantages of being less expensive and more accessible than MRI, its wide-scale use is limited by discordance between CAP and histology scores and a high technical failure rate (8%). Interestingly, and in contrast to other studies, the failure rate was independent of BMI in this study, although this may be because the average BMI was very high in this NAFLD population or because ‘failure’ may be simply an unreported or not attempted measure which we were unable to deduce in these data sets. Interestingly, we did observe a moderate correlation between BMI and CAP in the obese group (r = 0.23, P < .001) despite no correlation between BMI and steatosis grade (r_s_ = 0.1, P = .09). This is suggesting that in obese people in particular, CAP is confounded by body size. This is an important point, particularly due to gender difference in fat storage, as larger people with a favourable adiposity may be misdiagnosed as having steatosis. This could then impact decisions for their own health regarding suitability for other clinical interventions or their suitability as a liver donor.

MRI has been shown to provide a more accurate and comprehensive measure of liver health with techniques such as Liver *MultiScan*, which provide measures of liver iron, fibro-inflammation and liver fat [24,25,46]. Importantly, these measures are standardised across different MRI scanners and field strengths and have a very high technical success rate [47]. Further studies are needed to explore the prognostic value of these diagnostic techniques to determine the long-term outcomes of patients with NAFLD.

The large number of participants with biopsy-paired, non-invasive measures of liver fat is a strength of this study as larger cohort sizes lead to more accurate and representative results. However, pooling together participants from two independent studies may also have some limitations. Not only were the participants from different geographic populations (USA and Japan), they were also selected using two slightly different sets of criteria resulting in a pooled cohort with a mixture of liver health profiles. Study 1 only biopsied patients after a preliminary PDFF screening, which in NASH clinical trials is not an uncommon method to reduce screen fails at confirmatory biopsy, but potentially biases the study population by excluding some of the likely false negatives in the biopsy comparison. Whilst this is a limitation of the analysis, it was mitigated by pooling the data from the two studies. Furthermore, across the pooled cohort, MRI and CAP measurements were not all performed at the same centre, using the same MRI scanner models or ultrasound devices, or by the same operators. Another limitation is the use of liver biopsy as the reference for assessing steatosis, which although it remains the gold standard, is associated with sampling errors, as well as intra and inter-observer variability. In addition, in this pooled cohort, biopsies were not centrally read and whilst the combined sample size is substantial it does not contain a large number of those without steatosis; thus, linear relationships may not be accurately reflected. Some of these limitations could explain why the correlations between biopsy scores and LMS-IDEAL in the current study tend to be slightly lower than those reported in the literature. For example, the literature correlations between MRI-PDFF and histological scores ranged between r = 0.74 and r = 0.82 [16–18,20–22], compared with 0.54 in the current study. Notably, exploration of the two cohorts separately showed that the correlation between LMS-IDEAL and histology for study 1 was r = 0.58 and for study 2 was r = 0.71. It should also be noted that the presence of hepatic fibrosis has been reported to reduce the correlation between biopsy results and MRI PDFF, which was not accounted for in the current study [17,20].

### Conclusions

This is currently the largest study to demonstrate that MRI-based PDFF methods have higher diagnostic accuracy than CAP for detection of different levels of liver steatosis in patients with NAFLD. The results provide strong evidence that the MRI-based PDFF technique, LMS-IDEAL, is an excellent diagnostic and monitoring tool for NAFLD in both the clinical trial and in clinical practice settings.

## Supporting information

S1 Dataset Full study dataset. (CSV)
